# Potential Trace Metal Co-Limitation Controls on N_2_ Fixation and ${\text{NO}}_{3}^{-}$ Uptake in Lakes with Varying Trophic Status

**DOI:** 10.3389/fmicb.2013.00054

**Published:** 2013-03-20

**Authors:** I. C. Romero, N. J. Klein, S. A. Sañudo-Wilhelmy, D. G. Capone

**Affiliations:** ^1^Department of Biological Sciences, University of Southern CaliforniaLos Angeles, CA, USA; ^2^Department of Earth Sciences, University of Southern CaliforniaLos Angeles, CA, USA

**Keywords:** N_2_ fixation, **${\text{NO}}_{3}^{-}$** uptake, lake, trace metals, molybdenum

## Abstract

The response of N_2_ fixation and ${\text{NO}}_{3}^{-}$ uptake to environmental conditions and nutrient enrichment experiments in three western U.S. lake systems was studied (eutrophic Clear Lake; mesotrophic Walker Lake; oligotrophic Lake Tahoe). We tested the effect of additions of bioactive trace metals molybdenum as Mo(V) and iron (Fe) as well as phosphate (P), N_2_ fixation, ${\text{NO}}_{3}^{-}$, carbon (C) fixation, chlorophyll *a* (Chl*a*), and bacterial cell counts under both natural conditions and in mesocosm experiments. We found distinct background N_2_ fixation and ${\text{NO}}_{3}^{-}$ uptake rates: highest at Clear Lake (N_2_ fixation: 44.7 ± 1.8 nmol N L^−1^ h^−1^), intermediate at Walker Lake (N_2_ fixation: 1.7 ± 1.1 nmol N L^−1^ h^−1^; ${\text{NO}}_{3}^{-}$ uptake: 113 ± 37 nmol N L^−1^ h^−1^), and lowest at Lake Tahoe (N_2_ fixation: 0.1 ± 0.07 nmol N L^−1^ h^−1^; ${\text{NO}}_{3}^{-}$ uptake: 37.2 ± 10.0 nmol N L^−1^ h^−1^). N_2_ fixation was stimulated above control values with the addition of Fe and Pin Clear Lake (up to 50 and 63%, respectively); with Mo(V), Fe, and P in Walker Lake (up to 121, 990, and 85%, respectively); and with Mo(V) and P in Lake Tahoe (up to 475 and 21%, respectively). ${\text{NO}}_{3}^{-}$ uptake showed the highest stimulation in Lake Tahoe during September 2010, with the addition of P and Mo(V) (∼84% for both). High responses to Mo(V) additions were also observed at some sites for C fixation (Lake Tahoe: 141%), Chl*a* (Walker Lake: 54% and Clear Lake: 102%), and bacterial cell counts (Lake Tahoe: 61%). Overall our results suggest that co-limitation of nutrients is probably a common feature in lakes, and that some trace metals may play a crucial role in limiting N_2_ fixation and ${\text{NO}}_{3}^{-}$ uptake activity, though primarily in non-eutrophic lakes.

## Introduction

Nitrogen (N) plays a critical role in the productivity of many aquatic ecosystems. N occurs in a variety of inorganic forms in the environment (e.g., N_2_, ${\text{NH}}_{4}^{+}$, ${\text{NO}}_{3}^{-}$) and as various organic molecules in living cells (e.g., amino and nucleic acids). Although N is the most abundant element in the atmosphere, its bioavailability depends largely on a suite of transformations catalyzed primarily by microbes (Glass et al., 2009). Two of the most important processes in the N cycle of lakes are N_2_ fixation and ${\text{NO}}_{3}^{-}$ assimilation. Only certain prokaryotes (at a large energetic expense of 16 ATP) can fix N_2_ into 2NH_3_, which is subsequently assimilated through the Glutamine Synthetase-Glutamine OxoGlutarate Aminotransferase (GS – GOGAT) pathway (Meeks et al., 1978; Carpenter et al., 1992; Glass et al., 2009). Alternatively, both prokaryotes and some eukaryotes can enzymatically reduce ${\text{NO}}_{3}^{-}$ to ${\text{NO}}_{2}^{-}$ (Stolz and Basu, 2002) followed by reduction to ${\text{NH}}_{4}^{+}$which is also assimilated through GS – GOGAT (Muro-Pastor et al., 2005).

It has been postulated that cyanobacterial N_2_ fixation in lakes is largely controlled by dissolved inorganic N availability (Hyenstrand et al., 1998; Ferber et al., 2004). However, in many eutrophic lakes, it is the relative availability of phosphorus (P) and N which has generally been found to control N_2_-fixing populations (Schindler, 1977; Tilman et al., 1982), with substantial N_2_ fixation occurring where N is more severely limiting than P. However, other studies have found no relationship between N_2_ fixation and N:P levels (Toetz and McFarland, 1987; Smith, 1990). ${\text{NO}}_{3}^{-}$ assimilation is thought to be regulated primarily by light (for energy and as a reductant) and nutrients such as P and/or iron (Fe) (Dodds and Priscu, 1990; Tanigawa et al., 2002; Vasquez-Bermudez et al., 2003; Gardner et al., 2004; Nydick et al., 2004; Ivanikova et al., 2007).

Many of the key enzymatic reactions in the N cycle require not only major nutrients (e.g., P) but also trace metals such as Fe and molybdenum (Mo) for their synthesis and activity (Falkowski, 1983; Blanco et al., 1989; Dos Santos et al., 2004; Berges and Mulholland, 2008; Glass et al., 2009). Biological N_2_ fixation and ${\text{NO}}_{3}^{-}$ assimilation are catalyzed by Mo- and Fe-containing enzymes (nitrogenases and ${\text{NO}}_{3}^{-}$ reductases, respectively; Sigel and Sigel, 2002). Although non-Mo nitrogenases are known (Bishop and Joergert, 1990; Bishop and Premakumar, 1992), Mo-containing nitrogenases are predominant in most environments studied. However, more than a single trace element can limit different biochemical reactions (Saito et al., 2008), suggesting that co-limitation by trace metals, in addition to or in combination with some inorganic nutrients, can regulate biological processes.

In freshwater environments Fe is usually found at higher concentrations (in μM to mM concentration range; Vrede and Tranvik, 2006; Lofts et al., 2008; Warnken and Santschi, 2009) whereas Mo is generally very low (in the nM range; Cole et al., 1993; Magyar et al., 1993; Johannesson et al., 2000; Wang et al., 2009; Glass et al., 2012). The availability of Mo has been previously shown to be important in controlling primary productivity and ${\text{NO}}_{3}^{-}$ reduction in a meso-oligotrophic lake (Goldman, 1960; Axler et al., 1980; Glass et al., 2012) in contrast to P-sufficient lakes where Mo-additions have little effect on phytoplankton standing crop (Evans and Prepas, 1997). Thus, there is some evidence for a relationship between Mo availability and N_2_ fixation and ${\text{NO}}_{3}^{-}$ assimilation in lakes. However, in none of these studies was the speciation of Mo considered, and experimental Mo amendments were always made with the hypothetically less-bioavailable chemical form of Mo, as Mo(VI) molybdate (Howarth et al., 1988b). Co-limitation by reduced Mo [i.e., Mo(V)] may explain why N_2_ fixation often does not occur in oligotrophic lakes, despite the presence of potentially N_2_-fixing cyanobacteria (Ferber et al., 2004).

The main objective of our study was to determine major nutrient controls on N_2_ fixation and ${\text{NO}}_{3}^{-}$ uptake in three western U.S. lakes with varying trophic status (oligotrophic Lake Tahoe; mesotrophic Walker Lake; and eutrophic Clear Lake). We also studied nutrient controls on bacterial cell counts, chlorophyll *a* (Chl*a*), and carbon (C) fixation as proxies for bacterial growth, phytoplankton biomass, and total CO_2_ fixation, respectively. Although many factors can control the N cycle (e.g., wind, turbulence, temperature, grazing), here we concentrate on the nutrient that can regulate the biochemical reactions of the N_2_ fixation and ${\text{NO}}_{3}^{-}$ uptake pathways. While previous studies have shown the important role of Fe and P in the N cycle of lakes, the dynamics of these nutrients cannot always explain observed rates of N_2_ fixation (e.g., Axler et al., 1980; Ferber et al., 2004). Therefore, our study investigates not only Fe and P controls on N_2_ fixation and ${\text{NO}}_{3}^{-}$ uptake but also trace metal co-limitation, including the potentially most-bioavailable form of Mo, Mo(V). The results obtained in our study provide a new perspective on nutrient co-limitation in the N cycle of lakes.

## Materials and Methods

### Study sites

**Figure 1:**
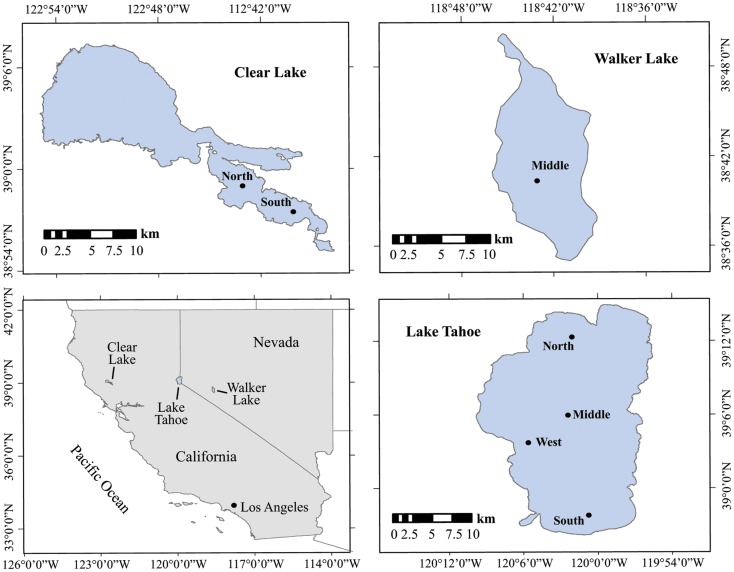
**Location and sampling stations of the lakes studied in California and Nevada**. Black dots represent the sampling stations.

The lakes studied are situated in California and Nevada, USA (Figure 1; Table 1). They were chosen to represent contrasting limnological conditions, algal assemblages, and diazotrophic populations with varying trophic status expected to influence the N cycling. Lake Tahoe (39°03′N, 122°48′W) is ultra-oligotrophic with the smallest forested watershed (800 km^2^), greatest maximum depth (505 m), and largest area (500 km^2^) of the lakes studied. It is located in the Sierra Nevada range at an elevation of 1898 m. Over the last several decades, human development in the Tahoe Basin has increased its primary productivity (5% annually), decreased its clarity (0.25 m y^−1^) and shifted it from a strongly N limited system toward more P limited as ${\text{NO}}_{3}^{-}$ in the lake has increased (Goldman, 1988, 2000; Reuter et al., 2003). N_2_ fixation research in Lake Tahoe has been focused on periphyton communities estimated to account for about 72% of the total dissolved inorganic nitrogen assimilated (Reuter et al., 1983). Walker Lake (39°05′N, 120°03′W) is a terminal monomictic and mesotrophic lake (Sharpe et al., 2008) with the lowest O_2_ concentration values of the lakes studied. It is located within a topographically closed basin in west-central Nevada at an elevation of 1300 m. Walker Lake is P rich and N limited. Thermal stratification occurs during summer with blooms of the cyanobacterium *Nodularia spumigena*, which dominates the phytoplankton (Horne et al., 1994). Clear Lake (38°41′N, 118°44′W) is eutrophic with the shallowest depth of the lakes studied. It is located in the northern Coastal Range of California at an elevation of 402 m. Previous limnological studies and N_2_ fixation measurements have been conducted in this lake since the early 1970s. N_2_ fixation is largely associated with cyanobacteria of the genera *Aphanizomenon* and *Anabaena* (Horne and Goldman, 1972).

**Table 1:** **General characteristics of the lakes studied (Lake Tahoe, Walker Lake, and Clear Lake)**.

Feature	Lake Tahoe	Walker lake	Clear lake
Surface area (km^2^)	500	130	170
Mean depth (m)	313	22	10
Max. depth (m)	505	26	18
Elevation (m)	1898	1300	402
Trophic status	Oligotrophic	Mesotrophic	Eutrophic
O_2_ (mg L^−1^)	9	8	9
Salinity (g kg^−1^)	0	17	0
TP (μM)	21 (10–40)	5030 (4729–5331)	275 (246–303)

*TP, total phosphorus*.

*Data shown as arithmetic mean (range)*.

### Field sampling

From 2009 to 2010 water was collected from the three lakes at different times with distinct limnological conditions likely to influence trace metal availability, as well as N_2_ fixation and ${\text{NO}}_{3}^{-}$ uptake. Sampling campaigns were conducted from April (late spring) to October (early fall) to capture periods of high abundance of phytoplankton and nutrients in the water column. Due to logistical constraints, most of our sampling effort was limited to the first 10 m depth (Lake Tahoe: October 2009, September 2010; Walker Lake: April 2010, September 2010; Clear Lake: July 2010). On several occasions we were able to obtain samples from >10 m depth (Lake Tahoe: October 2009; Walker Lake: October 2009).

Surface water samples were collected using a trace-metal-clean Teflon pumping system followed by in-line filtration through a 0.45 μm acid-washed polypropylene capsule filter. In Lake Tahoe, samples from depth profiles were also collected in September 2010 using a pre-programed rosette with trace-metal-clean Niskin bottles. Separately, hydrographic profiles were obtained using a YSI temperature/conductivity sensor (September 2010). The analyses of background water chemistry allowed us to establish the ambient levels of major and minor nutrients.

### Nutrient enrichment experiments

The potential limitation of C and N_2_ fixation, and ${\text{NO}}_{3}^{-}$ uptake by Mo(V), Fe, and/or P was assessed in 45 h incubations, relative to unenriched controls. Incubations for 24 h were conducted as well but no response was observed in any treatment or lake tested (data not shown). Total incubation volumes for the sampling campaigns were about 18 L (trace-metal-cleaned cubitainers) per treatment, including two to three replicates. All cubitainers were acid-cleaned (10% HCl) and rinsed with *in situ* water before use. We spiked with Mo(V) (see Wang et al., 2009 for details on solution preparation), Fe (as iron sulfate; FeSO_4_), and P (as monobasic potassium phosphate; KH_2_PO_4_). Final concentrations for the additions were 240–300 nMMo(V), 242–276 nM Fe, and 292–316 nM ${\text{PO}}_{4}^{-}$ which varied according to background concentrations measured previously and environmental conditions at the time of sampling in each lake (Table 2). Samples were incubated under ambient temperature conditions (Table 2) in a temperature-controlled incubator (Lake Tahoe and Walker Lake) or performed *in situ* with cubitainers suspended in the water column at an approximate depth of 0.5 m (Clear Lake). All samples for experiments were collected from the surface of the water column (0.5 m). Because incubation conditions varied among experiments conducted at each lake, treatment responses were normalized to the control (Ctrl) samples (see below for details). At the beginning (Ctrl treatment) and end (all treatments) of each experiment, samples for C and N metabolism, Chl*a*, and bacterial counts were taken from all incubation vessels and processed as described below.

**Table 2:** **Parameters measured at different stations during the sampling campaigns in Lake Tahoe, Walker Lake, and Clear Lake**.

	Lake Tahoe										Walker lake			Clear lake		
	October 2009								September 2010		April 2010		September 2010		June 2010	
	North	South	Middle			West			North	South		Middle	Middle		North	South
Depth (m)	0.5	0.5	0.5	50	70	0.5	50	70	0.5	0.5	0.5	20	0.5	0.5	10	0.5
Salinity (ppt)	0	0	0	0	0	0	0	0	0	0	22	22		0	0	0
Temp. (C)	14	14	14			14			21	19	12	11	21	23	20	23
TP (nM)	28	48	9.9 (0.20)	31 (8.1)	9.2 (1.2)	10 (1.5)	14 (1.2)	15 (1.2)	25 (1.0)	40 (0.80)	5300 (21)	5600 (27)	4700 (49)	250 (5.6)	910 (20)	300 (11)
${\text{PO}}_{4}^{3-}$ (μM)	0.77	1.1	0.80	0.82 (0.040)	0.85 (0.010)	0.83	0.85 (0.030)	0.85 (0.040)	0.86 (0.080)	1.0 (0.11)	25	27	23 (1.2)	0.30	2.1	0.48
${\text{NO}}_{3}^{-}$ (μM)	0.14		0.35	0.38	1.5				0.37 (0.17)	0.55 (0.040)	0.61 (0.030)	1.3 (0.010)	0.37 (0.41)	0.54	0.39	0.20 (0.13)
${\text{NO}}_{2}^{-}$(μM)	<LOD		0.026	0.020	<LOD				0.0059 (0.0048)	0	0.022 (0.11)	0.043 (0.046)	0.060 (0.0061)	0.037	0.041	0.0075 (0.0028)
C:N (mol)			12 (1.8)			8.9			8.3 (0.70)	8.5 (0.60)	9.9 (0.10)		11 (0.90)			
Total Mo (nM)	35.5	28.1	35.6 (0.102)	36.4 (1.77)	33.5 (0.696)	34.3 (0.535)	33.4 (0.536)	34.7 (0.633)	32.3 (0.102)	32.7 (0.128)	3020 (15.0)	3100 (27.3)	33300 (29.6)	3.16 (0.0230)	3.19 (0.0623)	3.44
Mo(V) (pM)	<LOD	<LOD	<LOD	514 (11.4)	512 (22.8)	<LOD	484 (30.2)		<LOD	<LOD	410 (283)	6190 (180)	<LOD	388 (10.8)	305 (8.71)	321 (11.1)
Mo(VI) (nm)	35.5	28.1	35.6	35.9	33.0	34.3	32.9	34.7	32.3	32.7	3020	3090	33300	2.77	2.89	3.12
Al (nm)	45	78	5.6 (0.61)	4.9 (2.6)	2.7 (5.9)	15 (5.4)	20 (6.2)	20 (2.2)	6.5 (0.10)	19 (0.16)	1400 (12)	2700 (3.7)	110 (1.1)	24 (0.47)	17	5300 (56)
Ti (nm)	0.050	1100	0.080 (0.0050)	0.12 (0.010)	0.030 (0.024)	0.18 (0.066)	0.26 (0.080)	0.34 (0.089)	0.25 (0.017)	1.6 (0.21)	22 (0.70)	25 (0.77)	17 (1.3)	3.1 (0.29)	2.4 (0.50)	15 (2.2)
V (nm)	13	9.0	13 (0.074)	12 (0.39)	11 (0.33)	12 (0.35)	12 (0.26)	12 (0.44)	12 (0.20)	12 (0.12)	96 (0.40)	96 (0.70)	98 (0.74)	23 (0.19)	20 (0.21)	23 (0.49)
Mn (nm)	4.7	10	0.78 (0.082)	0.34 (0.034)	0.25 (0.15)	1.2 (0.19)	0.42 (0.050)	0.39 (0.026)	1.9 (0.022)	9.2 (0.055)	36 (0.26)	36 (0.15)	14 (0.43)	52 (0.70)	38 (0.43)	35 (0.20)
Fe (nm)	18	150	2.3 (0.18)	1.6 (0.29)	0.91 (0.31)	9.4 (0.15)	14 (5.6)	18 (7.6)	4.1 (0.076)	120 (2.6)	150 (1.2)	110 (1.0)	92 (0.21)	110 (2.9)	74 (1.7)	180 (5.9)
Co (nm)	0.066	0.10	0.024 (0.0016)	0.021 (0.011)	0.011 (0.0036)	0.020 (0.0042)	0.026 (0.044)	0.027 (0.0060)	0.13 (0.0078)	0.12 (0.0080)	2.1 (0.046)	2.2 (0.25)	1.7 (0.12)	0.57 (0.10)	0.50 (0.16)	0.74 (0.21)
Ni (nm)	0.51	0	0.66 (0.067)	0.54 (0.16)	0.29 (0.016)	0.088 (0.083)	0.34 (0.11)	0.44 (0.11)	1.5 (0.16)	6.9 (0.21)	1.5 (0.056)	2.2 (0.053)	1.0 (0.061)	16 (0.084)	16 (0.41)	17 (0.28)
Cu (nm)	2.2	1.7	0.75 (0.10)	0.71 (0.4)	0.65 (0.38)	0.71 (0.38)	0.87 (0.11)	1.0 (0.16)	5.9 (0.047)	20 (0.42)	5.6 (0.15)	6.0 (0.07)	4.4 (0.14)	8.5 (0.22)	3.2 (0.30)	16 (0.023)
Cd (pm)	0.032	0.044	0.080 (0.0033)	0.056 (0.015)	0.042 (0.48)	0.052 (0.41)	0.060 (0.11)	0.056 (0.16)	0.039 (0.0012)	0.044 (0.0029)	3.4 (0.10)	3.5 (0.020)	3.8 (0.064)	0.024 (0.026)	0.023 (0.0012)	0.072 (0.05)
Ba (nM)	440	230	100 (35)	130 (13)	120 (7.4)	140 (24)	110 (1.2)	110 (4.5)	80 (1.1)	81 (0.90)	630 (0.11)	340 (0.13)	820 (0.12)	520 (4.1)	660 (13)	1100 (96)
pb (pm)	0.054	0.059	0.0075 (0.0016)	0.031 (0064)	0.0089 (0.0026)	0.011 (0.038)	0.088 (0.035)	0.074 (0.033)	0.13 (0.10)	0.44 (0.14)	0.69 (5.2)	0.56 (0.50)	0.18 (1.5)	0.14 (0.10)	0.47 (0.0016)	0.81 (0.0060)
${\text{NO}}_{3}^{-}$ uptake nmol C L^−1^ h^−^1			30.3 (0.629)			30.4			26.7 (0.924)	49.1 (16.8)	69.4 (15.3)		136 (18.7)			
N2 fixation nmol C L^−1^ h^−^1	0.0071 (0.012)	0.022 (0.025)	0.051 (0.088)			0.0020 (0.0031)			0.051 (0.044)	0.28 (0.24)	1.2 (0.088)		2.4 (1.2)	41 (2.6)		48 (3.1)
C Fixation nmol C L^−1^ h^−^1			0.44 (0.00042)			11			94 (15)	210 (72)	34 (4.4)		520 (61)			
Chla L^−1^	0.81 (0.68)		0.48 (0.15)	1.3 (0.20)	2.2 (0.15)	0.35 (0.01)	2.0 (0.11)	1.0 (0.045)	0.38 (0.02)	0.38 (0.066)	5.5 (0.075)		4.6 (0.65)	32 (7.3)		26 (9.5)
Bacteria (μg) (10^6^ cells L^−1^)	0.7 (0.14)		1.1 (0.12)	1.2 (0.11)	0.85 (0.022)	0.99	1.0	0.56	0.20 (0.57)	0.20 (0.036)	2.9		3.8 (0.22)	2.4		4.0

*Data shown as arithmetic mean (SE)*.

*<LOD, below the limit of detection (see Materials and Methods for details)*.

### Trace metals and nutrient analyses

Water samples were collected in the field following trace-metal-clean protocols, acidified to pH < 2 with 6 N quartz-distilled HCl (Optima-grade) and stored for at least a month prior to analysis (Sañudo-Wilhelmy and Flegal, 1996) by inductively coupled plasma-mass spectrometry (ICP-MS). By ICP-MS, we determined the total dissolved concentrations of total P (hereafter abbreviated TP) and trace metals (Al, Ti, V, Mn, Fe, Co, Ni, Cu, Cd, Ba, and Pb). The chemical speciation of molybdenum [Mo(V) and Mo(VI)] was measured according to the technique described by Wang et al. (2009). ICP-MS limits of detection (LOD) ranged from 0.8 pM for Cd to 250 pM for TP, and individual element concentrations for all samples were a minimum of one order of magnitude greater than their respective LODs. The LOD for Mo and Mo(V) was 2.8 pM.

Major nutrient samples were collected from the field (1–2 replicates per depth) and the nutrient enrichment experiments (three replicates per treatment), and stored at −20°C. ${\text{NO}}_{3}^{-}$ plus ${\text{NO}}_{2}^{-}$ were determined spectrophotometrically after reduction of ${\text{NO}}_{3}^{-}$ with spongy cadmium using a 10-cm optical path length (detection limit of 0.03 μM ${\text{NO}}_{3}^{-}$; Jones, 1984). ${\text{PO}}_{4}^{-}$ was determined spectrophotometrically with a 10-cm optical path (detection limit of 0.03 μM ${\text{PO}}_{4}^{-}$; Strickland and Parsons, 1972).

Water samples for particulate C and N analysis were collected from the field sampling (2–3 replicates per depth) and the nutrient enrichment experiments (three replicates per treatment), filtered (pre-combusted GF/F, 0.7-μm pore size) and stored frozen (−20°C). GF/F filters were dried for 24 h at 60°C. Particulate C and N analyses were performed on a mass spectrometer (see below) at the University of Southern California.

### Phytoplankton Chl*a* and bacterial counts

Water samples for phytoplankton Chl*a* and bacterial counts were collected from the field sampling (1–2 replicates per depth) and nutrient enrichment experiments (three replicates per treatment). Samples for phytoplankton Chl*a* (0.5–1.0 L) were collected and immediately filtered (pre-combusted GF/F, 0.7-μm pore size) and stored at −20°C. Fluorometric measurements of extracted Chl*a* were taken following established protocols (Arar and Collins, 1997).

Samples for bacterial counts (40 mL) were collected in sterile centrifuge tubes and stored at 4°C with 2% formalin (pre-filtered 0.02-μm pore size). Samples were processed for SYBR Green I microscopy following established protocols (Noble and Fuhrman, 1998; Patel et al., 2007). Slides (2 mL preserved sample) were observed using epifluorescence microscopy, and 10 microscopic fields were counted for each slide. Samples were counted in duplicate.

### C and N metabolism

Water samples for C and N metabolism were collected from the field sampling (2–3 replicates per depth) and the nutrient enrichment experiments (three replicates per treatment). N_2_ fixation was determined by the acetylene reduction approach (Montoya et al., 1996; Capone and Montoya, 2001). Briefly, water samples (100 mL) were incubated in 160 mL bottles with acetylene (20%) for ∼8 h, and ethylene concentration in the gas samples was determined by comparison with standards on a FID Shimadzu GC-9A gas chromatograph. The acetylene reduction rates calculated were converted to N_2_ fixation rates assuming the conversion factor of 4:1 (C_2_H_2_:N_2_; Montoya et al., 1996).

${\text{NO}}_{3}^{-}$ uptake was determined by N15O3− incorporation (Glibert and Capone, 1993) in 1-l polycarbonate bottles that were amended with N15O3− (99 atom%, Sigma-Aldrich/Isotec N15O3− was 4–12% of ${\text{NO}}_{3}^{-}$ background) and capped. Also, in the same incubation bottles, C fixation was determined by ^13^C-bicarbonate incorporation (Hamersley et al., 2011) by adding ${\text{NaH}}^{13}\text{C}{\text{O}}_{3}$ (99 atom%, Sigma-Aldrich/Isotec ${\text{NaH}}^{13}\text{C}{\text{O}}_{3}$ was 0.02–3% of dissolved inorganic background) into the capped 2-L and 1-L polycarbonate bottles. Twenty-four hour incubations were ended by filtration into pre-combusted GF/F filters (0.7-μm pore size) and stored at −20°C. Additional duplicate bottles were filtered immediately after adding the tracers as time zero controls. No significant change was observed in the isotopic composition of C and N of particulate matter immediately after tracer injection. GF/F filters were dried for 24 h at 60°C. Mass spectrometric analysis was performed on a Micromass IsoPrime mass spectrometer at the University of Southern California. Rates were calculated by isotope mass balance as described by Montoya et al. (1996). ${\text{NO}}_{3}^{-}$ uptake and C fixation rates were not measured in Clear Lake due to technical problems.

### Data analysis

Experimental responses to nutrient additions for C fixation, N_2_ fixation, ${\text{NO}}_{3}^{-}$ uptake, Chl*a*, and bacterial counts were normalized to the Ctrl values in each experiment to facilitate comparison among the lakes at each sampling campaign. Responses were calculated as: ((treatment mean−Ctrl mean) × (Ctrl mean)^−1^ × 100).

Linear regression analysis was used to examine the influence of *in situ* environmental parameters on C and N_2_ fixation, ${\text{NO}}_{3}^{-}$ uptake, Chl*a*, and bacteria counts in the lakes studied (JMP Software version 8.0, SAS Institute Inc., Cary, NC, USA). All data were transformed (log_10_) to stabilize the variance and normalize the distribution of each variable. Level of statistical significance was set to <0.05.

## Results

### Lake-specific environmental conditions

Sampling campaigns from April (late spring) to October (early fall) captured the periods of high abundance of phytoplankton and nutrients in the water column in the lakes studied. Distinct limnological conditions among the lakes were observed that were likely influenced trace metal availability, as well as biological activity in the surface waters (Table 2).

Lake Tahoe is ultra-oligotrophic and during this study it showed the lowest values among all lakes studied for concentrations of TP, nitrogen (${\text{NO}}_{3}^{-}$ + ${\text{NO}}_{2}^{-}$), Mo(V), and all other metals analyzed (Table 2). Spatial variability was observed in this lake, with higher concentrations of TP and some metals (Al, Ti, Mn, Fe, Co, Cu, and Ba) in the north and south stations. Although water temperature ranged from 14 to 20°C (October 2009 and September 2010, respectively), only ${\text{NO}}_{3}^{-}$, Ni, and Cu increased in September 2010 (Table 2).

Walker Lake showed the highest values for TP, ${\text{PO}}_{4}^{3-}$, ${\text{NO}}_{3}^{-}$, total Mo, Mo(V), Ti, V, Co, and Cd of the three lakes studied (Table 2). During April 2010, samples were collected after a storm which influenced the chemistry of the lake through strong mixing of the water column. This event could explain our finding of higher concentrations of ${\text{NO}}_{3}^{-}$ (39%), Mo(V) (100%), Al (92%), Mn (62%), Fe (41%) in April compared to September of 2010.

Although Clear Lake is eutrophic, we observed the lowest values among the lakes studied of ${\text{PO}}_{4}^{3-}$and total Mo (Table 2). A large bloom of diazotrophic cyanobacteria (*Lyngbya*, *Gloeotrichia*, *Aphanizomenon*, and *Anabaena*) and the non-diazotrophic cyanobacterium *Microcystis* was present during June 2010, potentially explaining the observed low values of these nutrients.

### Biological variability among lakes

**Figure 2:**
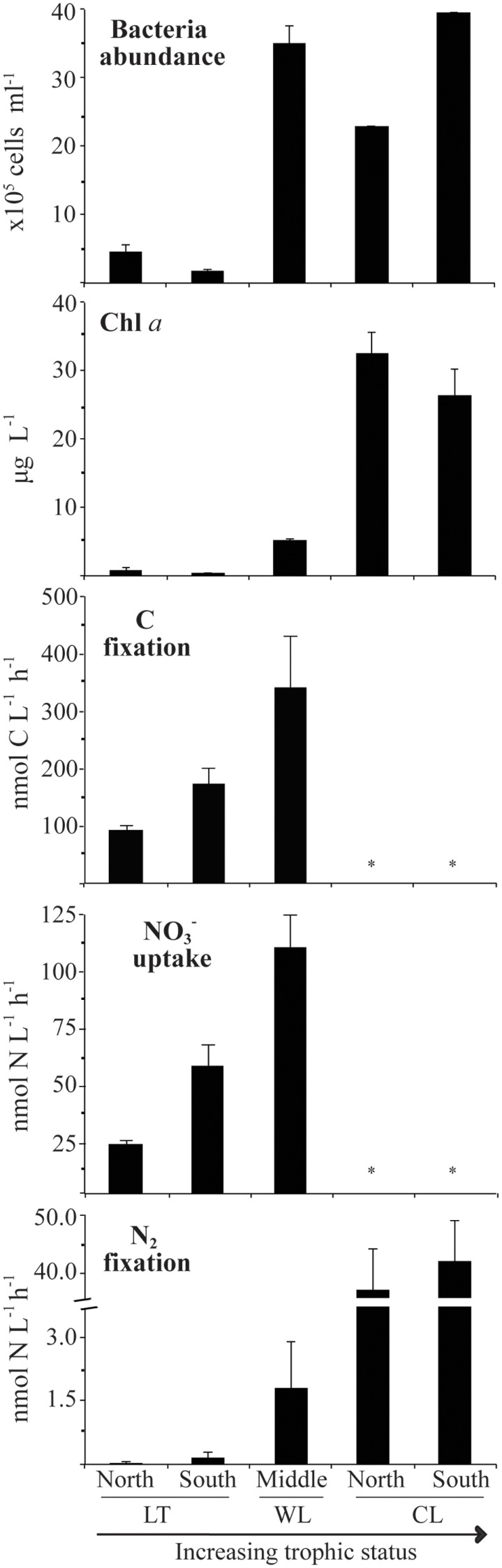
**Variability of biological parameters during the period studied from 2009 to 2010 (LT: Lake Tahoe; WL: Walker Lake; CL: Clear Lake)**. Measurements not determined denoted by *. Water samples were taken from 0.5 m below the surface. Data shown as arithmetic mean ± SE (*N* = 6–9).

As was expected, oligotrophic Lake Tahoe, having the lowest content of major and minor nutrients, also exhibited the lowest levels of biomass and biological activity (Table 2; Figure 2). On average bacteria abundance in Walker Lake (3.5 × 10^6^ cells L^−1^) and Clear Lake (3.2 × 10^6^ cells L^−1^) was 8× higher than in Lake Tahoe (0.4 × 10^6^ cells L^−1^). Walker and Clear Lakes also had higher Chl*a* concentrations (5.1 and 29.1 μg L^−1^, respectively) than Lake Tahoe (0.7 μg L^−1^) by 10× and 60×, respectively. The highest Chl*a* concentrations observed in Clear Lake (up to 35 μg L^−1^) were due to the large cyanobacterial bloom during the sampling campaign in June 2010 as noted above.

Background N_2_ fixation, ${\text{NO}}_{3}^{-}$ uptake, and C fixation rates differed among the lakes studied (Table 2; Figure 2). Lowest rates were observed at Lake Tahoe (N_2_ fixation: 0.10 ± 0.07 nmol N L^−1^ h^−1^; ${\text{NO}}_{3}^{-}$ uptake: 37.2 ± 10.0 nmol N L^−1^ h^−1^, C fixation: 79.5 ± 18.9 nmol C L^−1^ h^−1^). Intermediate rates were recorded at Walker Lake (N_2_ fixation: 1.7 ± 1.1 nmol N L^−1^ h^−1^; ${\text{NO}}_{3}^{-}$ uptake: 114 ± 37 nmol N L^−1^ h^−1^, C fixation: 340 nmol ± 90.9 C L^−1^ h^−1^). The highest N_2_ fixation rates were noted at Clear Lake (N_2_ fixation: 44.7 ± 1.8 nmol N L^−1^ h^−1^). Among all measured variables, N_2_ fixation showed the largest differences among the lakes with rates at Walker Lake and Clear Lake 20× and 490× higher (respectively) than in Lake Tahoe. In contrast, ${\text{NO}}_{3}^{-}$ uptake and C fixation were only 2–4× higher in Walker Lake than in Lake Tahoe.

### Responses to nutrient enrichment experiments

Bacterial cell growth was stimulated above controls by all nutrient enrichment treatments in five out of seven experiments independent of the trophic status of the lakes (Table 3). Chl*a* was stimulated above control values by Mo(V) and P in Walker Lake in September 2010 (54 and 9%, respectively) and Clear Lake (north station: 102 and 22%, respectively). Fe was stimulatory only once, in June 2010 at Clear Lake at the northern station. Slight stimulation of Chl*a* occurred on three occasions in Lake Tahoe (October 2009, north station by Mo(V) and P; Sept 2010, south station by P).

**Table 3:** **Normalized responses (%) of biological parameters to nutrient additions [Mo(V), Fe, P] observed in the three lakes studied at different times**.

		Bacteria cells			Chl *a*			C fixation			${\text{NO}}_{3}^{-}$ uptake			N_2_ fixation		
Lake/date	Site	Mo (V)	Fe	P	Mo (V)	Fe	P	Mo (V)	Fe	P	Mo (V)	Fe	P	Mo (V)	Fe	P
**LAKE TAHOE**																
October 2009	North	62	87	50	11	−18	11	−64	6	−80	−6	−42	2	281	−55	−1
	South													475	−25	21
September 2010	North	25	40	84	−10	−15	−14	12	−2	26	−2	−8	11	−6	−3	−21
	South	−39	34	9	−7	−4	3	141	138	127	83	−30	85	345	−65	−62
**WALKER LAKE**																
April 2010	Middle	36	54	43	−20	−6	−8	4	4		21	−66		121	169	11
September 2010	Middle	29	31	10	54	−4	9	−52	−44	−48	−11	−14	−18	−65	989	86
**CLEAR LAKE**																
June 2010	North	−34	−16	23	102	105	22							13	6	41
	South	1	93	124	−22	−8	29							−12	51	62

*Values >0 indicate stimulation relative to controls, while values <0 represent suppression relative to controls*.

C fixation was stimulated above control values by Mo(V), Fe, and P in Lake Tahoe in September 2010 at the southern station (141, 138, and 127%, respectively). Slight stimulation by Mo(V) and P occurred at the northern station on this date. ${\text{NO}}_{3}^{-}$ uptake was also stimulated above control values with the addition of Mo(V) and P at the southern station in Lake Tahoe (83% up to 85%, respectively), and by Mo(V) in Walker Lake (only in April: 21%). Strong stimulation of N_2_ fixation above control values was observed for Mo(V) in Lake Tahoe in both October 2009 and September 2010 for the southern station (up to 475%) and only in October 2009 for the northern station, and in April 2010 for Walker Lake (121%). Fe and P also stimulated N_2_ fixation rates above control values in Walker Lake (up to 989 and 86%, respectively) and Clear Lake (up to 51 and 63%, respectively).

### Nutrient limitation controls on biological processes

Correlation analysis between biological parameters and *in situ* major and minor nutrient concentrations revealed significant positive correlations between ambient ${\text{PO}}_{4}^{-}$ and/or metals and bacterial counts, Chl*a*, ${\text{NO}}_{3}^{-}$ uptake, and N_2_ fixation but not C fixation (Table 4). Bacterial cell counts were significantly positively correlated to Al, Ti, V, Co, and Ba and not to nutrients used in the enrichment experiments (Table 4; *P* < 0.05). Chl*a* was also significantly positively correlated to Al, Ti, and Co, and, also to Mn and the calculated ratio Mo(V):${\text{PO}}_{4}^{-}$ (Table 4; *P* < 0.05). A relationship between Chl*a* and nutrients such as Mo(V) and ${\text{PO}}_{4}^{-}$ was also observed in the enrichment experiments in Tahoe Lake (October 2009), Walker Lake (only during September 2010 when nutrient concentrations were low), and Clear Lake (primarily at the northern station where concentration of nutrients were high; Tables 2 and 3). ${\text{NO}}_{3}^{-}$ uptake was significantly positively correlated to ${\text{PO}}_{4}^{-}$, Mo(VI), Ti, V, Co, Cd, Ba, and Mo(VI): ${\text{PO}}_{4}^{-}$ (Table 4; *P* < 0.05). A relationship between ${\text{NO}}_{3}^{-}$ uptake and ${\text{PO}}_{4}^{-}$ was also observed in the enrichment experiments in Lake Tahoe where ${\text{PO}}_{4}^{-}$ is low (Tables 2 and 3). N_2_ fixation was significantly positively correlated to Mo(V), Mn, Ni, Ba, and the calculated ratios Mo(V):Fe and Mo(V): ${\text{PO}}_{4}^{-}$ (Table 4; *P* < 0.05). A relationship between N_2_ fixation and nutrients such as Mo(V), Fe, and ${\text{PO}}_{4}^{-}$was also observed in the enrichment experiments with different responses in each lake (Table 3).

**Table 4:** **Correlation analysis between biological parameters and *in situ* nutrient conditions**.

	Bacteria cells			chl*a*			C fixation			${\text{NO}}_{3}^{-}$ Uptake			N_2_ fixation		
	*R*	*P*-value	*n*	*R*	*P*-value	*n*	*R*	*P*-value	*n*	*R*	*P*-value	*n*	*R*	*P*-value	*n*
${\text{PO}}_{4}^{3-}$	0.448	0.125	13	0.124	0.685	13	0.431	0.394	6	0.882	0.020*	6	−0.111	0.761	10
${\text{NO}}_{3}^{-}$	−0.096	0.793	10	−0.016	0.966	10	0.210	0.734	5	0.247	0.689	5	0.006	0.990	8
${\text{NO}}_{2}^{-}$	0.639	0.047	10	0.386	0.270	10	0.114	0.856	5	0.738	0.155	5	0.272	0.515	8
Mo(V)	0.399	0.199	12	0.496	0.101	12	−0.045	0.933	6	0.294	0.572	6	0.766	0.010*	10
Mo(VI)	0.245	0.442	12	−0.179	0.579	12	0.413	0.415	6	0.885	0.019*	6	−0.376	0.284	10
Al	0.622	0.023*	13	0.632	0.020*	13	0.311	0.549	6	0.711	0.113	6	0.537	0.110	10
Ti	0.684	0.010*	13	0.679	0.011*	13	0.526	0.284	6	0.911	0.012*	6	0.569	0.086	10
V	0.684	0.010*	13	0.486	0.092	13	0.406	0.425	6	0.882	0.020*	6	0.305	0.391	10
Mn	0.519	0.069	13	0.763	0.002*	13	0.589	0.219	6	0.798	0.057	6	0.762	0.010*	10
Fe	0.406	0.168	13	0.618	0.054	13	0.664	0.150	6	0.795	0.059	6	0.530	0.115	10
Co	0.683	0.010*	13	0.595	0.032*	13	0.448	0.374	6	0.861	0.028*	6	0.423	0.223	10
Ni	0.233	0.444	13	0.733	0.004*	13	0.534	0.275	6	0.171	0.747	6	0.851	0.002*	10
Cu	0.043	0.890	13	0.491	0.088	13	0.754	0.084	6	0.330	0.523	6	0.578	0.080	10
Cd	0.534	0.060	13	0.225	0.461	13	0.420	0.407	6	0.892	0.017*	6	0.004	0.990	10
Ba	0.747	0.003	13	0.594	0.052	13	0.341	0.508	6	0.867	0.025*	6	0.769	0.009*	10
Fe: ${\text{PO}}_{4}^{3-}$	0.128	0.676	13	0.596	0.082	13	0.363	0.480	6	−0.029	0.957	6	0.643	0.045	10
Mo(V):Fe	−0.056	0.863	12	−0.099	0.761	12	−0.045	0.933	6	0.294	0.572	6	0.762	0.011*	10
Mo(V): ${\text{PO}}_{4}^{3-}$	0.367	0.241	12	0.686	0.014*	11	−0.045	0.933	6	0.294	0.572	6	0.928	0.000*	10
Mo(VI):Fe	0.124	0.702	12	−0.340	0.279	12	−0.077	0.885	6	0.464	0.354	6	−0.393	0.261	10
Mo(VI): ${\text{PO}}_{4}^{3-}$	−0.014	0.965	12	−0.508	0.092	12	0.319	0.537	6	0.853	0.031*	6	−0.641	0.046	10

**R*, linear correlation coefficient*.

**P*-value, level of statistical significance at *P* < 0.05*.

**N*, number samples*.

*Significant correlation coefficient.

## Discussion

We studied C and N metabolism in three western U.S. lake systems between late spring and early fall to capture periods of high abundance of phytoplankton and nutrients in the water column. The lakes studied were characterized by different levels of biological activity (Table 2; Figure 2) each with a distinct trophic status (eutrophic Clear Lake; mesotrophic Walker Lake; oligotrophic Lake Tahoe) as reported previously (e.g., Wurtsbaugh, 1983; Sharp, 2009; Winder, 2009). Upper water column integration (surface to 5 m) of biological activity was calculated over all stations and months studied for Lake Tahoe (C fixation: ∼9.5 mmol C m^−2^ days^−1^; N_2_ fixation: ∼0.01 mmol N m^−2^ days^−1^; ${\text{NO}}_{3}^{-}$ uptake: ∼4.1 mmol N m^−2^ days^−1^), Walker Lake (C fixation: ∼33.4 mmol C m^−2^ days^−1^; N_2_ fixation: ∼0.2 mmol N m^−2^ days^−1^; ${\text{NO}}_{3}^{-}$ uptake: ∼12.3 mmol N m^−2^ days^−1^), and Clear Lake (N_2_ fixation: ∼5.4 mmol N m^−2^ days^−1^). Integrated rates are within the values reported in other lakes for N_2_ fixation (Howarth et al., 1988a; Ferber et al., 2004), ${\text{NO}}_{3}^{-}$ uptake (Ferber et al., 2004; Kumar et al., 2008; Rojo et al., 2008), and C fixation (Kumar et al., 2008; Piehler et al., 2009; Salm et al., 2009). High N_2_ fixation rates in Clear Lake were associated with cyanobacterial blooms as observed previously by Horne and Goldman (1972). There are no previously published N_2_ fixation rates for Walker Lake and Lake Tahoe for the water column. In Lake Tahoe, studies have focused primarily on periphyton communities showing high rates for N_2_ fixation and ${\text{NO}}_{3}^{-}$ uptake due to a large supply of nutrients through microbial remineralization of settled particles (Reuter et al., 1983). In Lake Tahoe, Winder (2009) reported C fixation rates for the upper 60 m water column during July to November (2–3 mmol C m^−2^ days^−1^).

We observed large biological variability in stations sampled during different campaigns as well as in the responses to nutrient additions in our experiments. Experimental responses to Mo(V) additions were positive (stimulation) for N_2_ fixation and negative (suppression) for C fixation only during the colder months, while bacterial cell counts showed positive responses during all months studied (Table 3). Temporally contrasting responses were also observed in experimental P additions for Chl*a*, and C and N_2_ fixation (Table 3). Changes in temperature and nutrients, as well as community composition in the water column may be the main factors contributing to the biological variability in our study. Multiple limiting nutrients, competition between different planktonic groups for the same pool of nutrients, stronger limitation by nutrient availability for phytoplankton than bacterioplankton, and seasonal changes in limiting nutrients appear to be common in lakes and other in-land systems (Ahmad, 1981; Marcarelli and Wurtsbaugh, 2006; Kumar et al., 2008; Salmaso, 2011). Thus, our observations show that multiple factors influence the response of planktonic communities to nutrient enrichment.

Overall, we found P was limiting phytoplankton C fixation or biomass accumulation (Chl*a*) in all lakes as well as ${\text{NO}}_{3}^{-}$ uptake in Lake Tahoe and N_2_ fixation in Walker Lake and Clear Lake (Table 3). Also, C fixation and N_2_ fixation were limited by Fe in Walker Lake and Clear Lake. Additionally, we observed that Mo(V) was limiting C fixation, N_2_ fixation, and ${\text{NO}}_{3}^{-}$ uptake in some experiments in Tahoe Lake and Walker Lake, and only Chl*a* in Walker Lake and Clear Lake (Table 3). Also, significant correlations between Mo(V): ${\text{PO}}_{4}^{3-}$ and Chl*a* and N_2_ fixation, and Mo(VI): ${\text{PO}}_{4}^{3-}$ and ${\text{NO}}_{3}^{-}$ uptake were observed using *in situ* data. In lake systems it is well known that P alone and in combination with other nutrients (e.g., N, Fe) often limits primary production and other processes like N_2_ fixation and ${\text{NO}}_{3}^{-}$ uptake (Horne and Goldman, 1972; Howarth et al., 1988b; Ferber et al., 2004; Lewis and Wurtsbaugh, 2008; Sterner, 2008). However, other studies have found no relationship between N_2_ fixation and N and P levels (Toetz and McFarland, 1987; Smith, 1990), leaving open the possibility of limitation by some factors such as Mo availability, which is known to be required by N_2_-fixing cyanobacteria for optimal rates of activity and growth (Glass et al., 2012 and references therein). Therefore, our study supports the notion that nutrient co-limitation of chemical transformations occurring in the N cycle and phytoplankton biomass and/or activity in lakes is common, and also suggests the importance of limitation by trace metals such as the reduced form of Mo, Mo(V).

The hypothesis regarding the involvement of Mo in the N cycle of lakes is not new (Glass et al., 2012 and references therein). Goldman (1960) demonstrated Mo deficiency in Castle Lake using a bioassay approach. Subsequent work in this lake linked Mo limitation to ${\text{NO}}_{3}^{-}$ reduction, since the addition of Mo was most effective at increasing primary production during periods of high ${\text{NO}}_{3}^{-}$ availability (Axler et al., 1980). In a study of 13 saline Alberta lakes, Marino et al. (1990) found that a low sulfate:Mo ratio was the best predictor of abundant populations of diazotrophic cyanobacteria. Wurtsbaugh (1988) reported that Mo could be a factor limiting N_2_ fixation in some lakes and reservoirs in the Great Salt Lake Basin. In contrast, Evans and Prepas (1997) surveyed a series of 11 apparently *P*-sufficient prairie saline lakes for controls on phytoplankton standing crop and found Mo-additions to have little effect in these systems. Thus, these studies reveal that a stronger connection between Mo cycling and N_2_ fixation occurs in more nutrient-deficient lakes.

**Figure 3:**
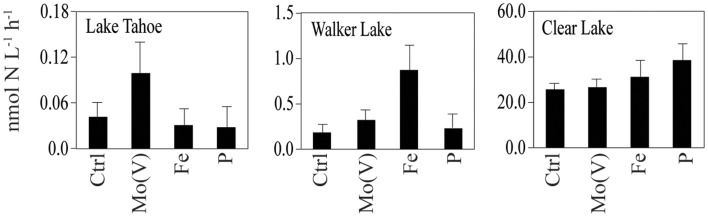
**N_2_ fixation measured in different lakes under different nutrient enrichment conditions [Ctrl, Mo(V), Fe, P] during the sampling campaigns of 2009 and 2010**. Water for incubation experiments was taken from a depth of 0.5 m. Data shown as arithmetic mean ± SE (*N* = 4–6).

Similarly, in our study we found Mo [specifically Mo(V)] to be a potentially limiting nutrient for N_2_ fixation primarily in nutrient-deficient Lake Tahoe (Figure 3). Consistent with this hypothesis, Mo-additions in Mo-enriched lakes like Walker Lake did not have any effect on N_2_ fixation (Figure 3). However, Clear Lake does not fit our hypothesis, as we observed the lowest Mo concentration (∼3.5 nM) and low response to Mo(V) additions (Table 3). Based on a comparison of dissolved concentrations at surface vs. depth (unpublished data), the sediments of Clear Lake do not appear to be a source of Mo to the water column, while total *P* is elevated more than threefold at depth. It is possible that the low concentrations of Mo observed in Clear Lake are a result of intensive uptake and recycling by the large biomass of cyanobacteria (due to blooms of *Lyngbya*, *Microcystis*, *Gloeotrichia*, *Aphanizomenon*, and *Anabaena*) coupled with a lack of sediment inputs of Mo to the lake system. Were this the case, it should be reflected by large particulate concentrations of Mo during the bloom and by higher dissolved Mo concentrations in the lake when a bloom was not occurring. Therefore, although further research is needed to understand the temporal and spatial variability of Mo limitation in lakes, and the effect of algal blooms on the relationship between Mo and N_2_ fixation (e.g., Clear Lake), our results provide confirmation that primary production, ${\text{NO}}_{3}^{-}$ uptake and N_2_ fixation can be limited by Mo availability in non-eutrophic lakes with low Mo levels. In addition, further evaluation of other metals limitation and/or co-limitation on biological activity should be considered as some bioactive elements (e.g., Co, Mn, and Ni) also showed a significant correlation with ${\text{NO}}_{3}^{-}$ uptake and/or N_2_ fixation (Table 4).

While Mo exists as a relatively unreactive oxyanion in the VI oxidation state in seawater (Bruland and Lohan, 2004), thermodynamic calculations show that in freshwater, 30–50% of the dissolved Mo should be present in the V oxidation state – under oxic and suboxic conditions respectively. In fact, the Eh range for the Mo(VI)/Mo(V) couple in freshwater (0.04–0.55 V, depending on the presence of organic ligands) is within the range of potentials reported for As, Mn, and Fe (Turner et al., 1981), metals whose reduced forms have been reported in many lacustrine environments (Anderson and Bruland, 1991; Davison, 1993). These results are not surprising as several ubiquitous mechanisms could result in the formation of Mo(V) species, such as reduction by organic acids and microbial activity (Szilágyi, 1967; Lovley, 1993; Lloyd, 2003). However, these results are based on laboratory experiments and the cycling of the different species of Mo in natural waters is still unknown. Another environmental factor that could influence the concentrations and speciation of dissolved Mo in lakes is seasonal anoxia. This is because under anoxic conditions, Mo can be reduced from the VI oxidation state to the insoluble MoS_2_(s) or converted to particle-reactive thiomolybdates (Vorlicek and Helz, 2002). Seasonal anoxia in stratified lakes could potentially reduce the total concentrations of dissolved Mo available to phytoplankton but increase the relative abundance of the reduced form of Mo [e.g., Mo(V)].

Our study provides evidence that Mo(V) stimulates N_2_ fixation at Lake Tahoe and to a lesser extent at Walker Lake and Clear Lake. While previous investigators have shown that Mo limitation of N_2_ fixation could result from sulfate inhibition of Mo uptake (Howarth et al., 1988a; Cole et al., 1993), they have also established that freshwater molybdate (VI oxidation state) uptake should be inhibited by only 1–5% by the sulfate levels found in most lakes (Marino et al., 1990, 2003). Therefore, as reported for other trace elements (Donat and Bruland, 1995), we hypothesize that Mo availability supporting N_2_ fixation and ${\text{NO}}_{3}^{-}$ uptake in lakes is mostly controlled by the chemical speciation of this element. The reduced forms of Mo [such as Mo(V)] are likely more bioavailable than Mo(VI), as the biological uptake of the reduced Mo should not be affected by the presence of inhibitors such as sulfate. This hypothesis is consistent with our results and recent studies showing that reduced species of Mo are found in nitrogenases (Howard and Rees, 2006) and in ${\text{NO}}_{3}^{-}$ reductases (González et al., 2006). Although further research is needed to understand the temporal and spatial variability of Mo limitation in lakes, the results obtained in our study provide a new perspective on trace metal limitation in lake systems.

## Conflict of Interest Statement

The authors declare that the research was conducted in the absence of any commercial or financial relationships that could be construed as a potential conflict of interest.
